# Prevalence of *Helicobacter pylori vacA*, *cagA* and *iceA* genotypes and correlation with clinical outcome

**DOI:** 10.3892/etm.2012.704

**Published:** 2012-09-11

**Authors:** GUO-CHAO WEI, JING CHEN, AI-YUN LIU, MIAO ZHANG, XIAO-JUN LIU, DAN LIU, JUN XU, BING-RONG LIU, HONG LING, HUA-XING WU, YA-JU DU

**Affiliations:** 1 Department of Gastroenterology, The Second Affiliated Hospital of Harbin Medical University;; 2 Department of Microbiology, Harbin Medical University;; 3 Department of Endoscopic Center, The Third Affiliated Hospital of Harbin Medical University, Harbin, Heilongjiang 150086, P.R. China

**Keywords:** *Helicobacter pylori*, prevalence, genotype, Northeast China

## Abstract

The aim of this study was to assess the genetic status of *cagA*, *vacA* subtype and *iceA* genotypes of *Helicobacter pylori* and the relationship with upper gastrointestinal diseases in Northeast China. Gastric biopsies were obtained from 378 patients with upper gastrointestinal diseases and 197 samples were used. The *cagA*, *vacA* alleles and *iceA* genotypes were determined by polymerase chain reaction. *CagA* was present in 176 (89.3%) of 197 patients. Of the 197 cases, 186 (94.4%) had *vacA* signal sequence s1c allele, 6 (3%) had s1a and 5 (2.5%) had s1b. The *vacA* s2 genotype was not detected in our study. *VacA* middle region sequences, m1 and m2, were found in 20 (10.2%) and 150 (76.1%), respectively. The allelic variant *iceA1* (70.1%) was more prevalent than *iceA2* (23.4%). The *vacA* allele s1am2 had a significant relationship with the presence of gastric cancer (p<0.05) and the *iceA1* genotype was also associated with gastric cancer (p<0.05). These may be useful risk factors for upper gastrointestinal diseases.

## Introduction

*Helicobacter pylori* (*H. pylori*) is a gram-negative microaerophilic bacterium which is one of the most common pathogens in humans and has a worldwide distribution. It is associated with the development of chronic gastritis, peptic ulcer and even gastric cancer (1). On the basis of abundant epidemiological research, *H. pylori* was classified as a class I carcinogen in humans by the World Health Organization International Agency for Research on Cancer (2).

Several *H. pylori* virulence genes that may be associated with the risk of developing diseases have been identified. The *cagA* is a marker of genomic pathogenicity island (cagPAI) encoding the gene product which causes upregulation of interleukin-8 (IL-8) (3). It is considered that *H. pylori* strains possessing *cagA* are related to a more severe clinical outcome such as atrophic gastritis or gastric cancer (4,5). The *vacA* exists in all *H. pylori* strains and encodes vacuolating cell toxins which cause vacuole degeneration of epithelial cells. It includes two different parts: the signal (s) region encoding the signal peptide and the middle (m) region. The s-region is situated at the 5′ end of the gene and exists as s1 and s2 alleles. The s1 exists as an s1a, s1b and s1c. The m-region occurs as m1 or m2 alleles (6). The mosaic combination of s- and m-region allelic types produces cytotoxin and is associated with the pathogenicity of the bacterium. In general, type s1m1 and s1m2 strains produce high and moderate levels of toxin, respectively, whereas s2m2 strains produce little or no toxin. (7). *VacA* m1 strains are associated with greater gastric epithelial damage than m2 strains (8). Another virulence gene designated *iceA* has two main allelic variants *iceA1* and *iceA2* but the function of these variants is unknown. *IceA1* is upregulated upon contact of *H. pylori* with the gastric epithelium and has been considered as a marker for peptic ulcer disease (9).

In Northeast China, there are no data regarding the pattern of *H. pylori* genotypes in patients. This study aimed to investigate the prevalence of the *vacA*, *cagA* and *iceA* genotypes of *H. pylori* from patients with upper gastrointestinal diseases and the relationship with clinical outcome in Northeast China.

## Materials and methods

### Study subjects

We evaluated 378 patients with upper gastrointestinal diseases referred for endoscopy at the Second Affiliated Hospital of Harbin Medical University in 2007 and 2008. Gastric mucosal biopsy specimens were obtained from each patient: one for pathological diagnosis, another for histological detection of *H. pylori* and the last for genomic DNA extraction and polymerase chain reaction (PCR).

The study was approved by the Ethics Committee of Harbin Medical University. Written informed consent was obtained from each patient prior to enrolling in the study.

### Histological assessment

The biopsy samples were fixated in 10% formalin, then sliced into 4- to 6-mm pieces, dehydrated in ethanol, embedded in paraffin wax, sectioned (5-*μ*m thick), and stained with hematoxylin and eosin (H&E). The presence of *H. pylori* in the sections was determined using a modified Gram staining protocol and taking into consideration its morphological characteristics which included a curved and spiral form and intense purple coloring (10). Pathological diagnoses were evaluated in a blinded manner by two independent pathologists and were defined as gastritis (active chronic gastritis or closed-type atrophic gastritis), gastric ulcer and gastric cancer.

### Genomic DNA extraction

DNA was extracted from the biopsy specimens using the Genomic DNA purification system (Promega, USA) according to the manufacturer’s instructions and stored at −20°C until analysis.

### *Diagnosis of H. pylori* infection

*H. pylori*-positive status was defined as positive histology and positive 16S-rRNA PCR. A 500-bp region of 16S-rRNA was amplified by PCR using primers CP-1/CP-2 (Table I). Five microlitres of DNA was added to 50 *μ*l of reaction mixture containing 1X PCR buffer, 0.2 mM dNTPs and 0.3 *μ*M primers as well as 1.25U Taq polymerase (Takara Bio, Inc., Japan). The incubation conditions were as follows: a 5-min preincubation at 95°C, followed by 30 cycles of 1 min at 94°C, 1 min at 58°C, 1 min at 72°C, and a final 5-min incubation at 72°C. Positive results were indicative of a diagnosis of *H. pylori* infection.

### Genotyping of H. pylori

The systems of PCR were the same as mentioned above except for the primers. The amplification cycles consisted of an initial denaturation at 94°C for 5 min and then denaturation at 94°C for 30 sec, primer annealing at 60, 56, 58 and 48°C for *cagA*, *vacA* (s1a, s1b, s1c and s2), *vacA* (m1, m2) and *iceA,* respectively, for one-half minute and extension at 72°C for 45 sec. All reactions were performed through 35 cycles. The final cycle included an extension step for 5 min. Primers used for genotyping *cagA*, *vacA* and *iceA* genes are listed in Table I. PCR products were analyzed on 1.5% agarose gel electrophoresis with ethidium bromide. Images were quantified via the Gene Genius system (Syngene, England, UK). For strains that were *cagA*-negative as determined by PCR, Southern blotting was performed according to the method described by Pan *et al* (11).

### Statistical analyses

Statistical tests were performed with SPSS software version 11.5 (SPSS Inc., Chicago, IL, USA). A Chi-square test and Fisher’s exact test were used to assess the association amongst the genotypes and between specific genotypes and upper gastrointestinal diseases. P-values <0.05 were considered to indicate a statistically significant result.

## Results

DNA was successfully extracted from 378 gastric mucosa tissues of patients with gastrointestinal diseases and 197 were confirmed as *H. pylori* infection-positive by histology and PCR amplification. *H. pylori*-infected patients were evaluated for the relationship of age and gender with disease as shown in Table II.

### *Detection of H.* pylori genotypes

**Figure 1:**
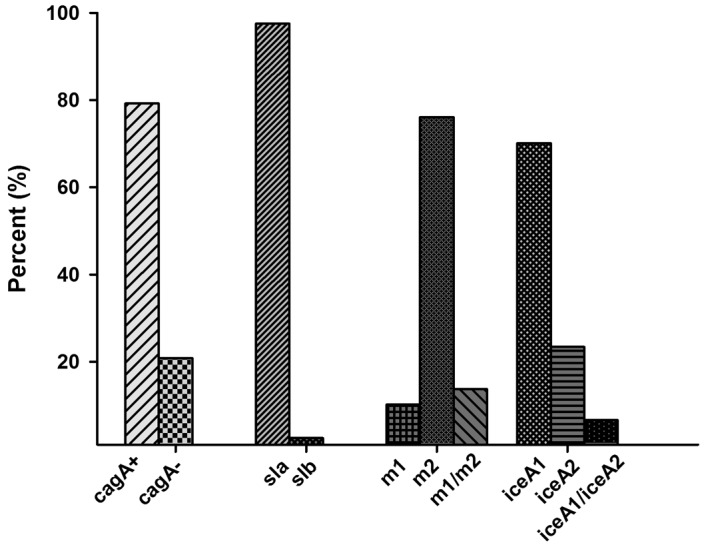
Distribution of *cagA*, *vacA* and *iceA* alleles of *H. pylori* from 197 patients with upper gastrointestinal diseases. M1m2, multiple *vacA* genotypes with m1 and m2. *IceA1*/*iceA2*, mixed *iceA* genotypes with *iceA1* and *iceA2*.

Overall, the presence of the *cagA* gene was detected in 176 cases (89.3%). A negative status for the other 21 (10.7%) cases was confirmed by Southern blotting, and the results were negative as before. All of the samples were positive for *vacA* (both the s-region and the m-region). Of the 197 cases, 186 (94.4%) had *vacA* signal sequence s1c allele, 6 (3%) had s1a and 5 (2.5%) had s1b. The *vacA* s2 genotype was not detected in our study. In the m-region, 27 cases contained both m1 and m2. In these cases the m1 allele was found in 20 (10.2%) isolates and m2 (76.1%) in 150 cases, which indicating the presence of mixed infection. The *vacA* s1am2 genotype was identified in 6 (3.0%) participants, the *vacA* s1bm2 was identified in 5 (2.5%) participants, s1cm1 was identified in 20 participants, and s1cm2 gene was identified in 139 ones. *IceA1* was found in 138 (70.1%) and *iceA2* was detected in 46 (23.4%) cases. The *iceA2* amplification yielded both the 229-and 334-bp bands due to the presence of a 105-bp in-frame amplicon present in the 334-bp band that was absent in the 229-bp band. Mixed *iceA* (*iceA1* + *iceA2*) genotypes were found in 13 (6.7%) of our isolates (Fig. 1).

### Association among the genotypes

*CagA* was present in 124 out of 138 *iceA1* cases (91.1%) and 44 out of 46 *iceA2* cases (95.5%) (p>0.05) where 29 patients with mixed infection were excluded. Due to the lack of *vacA* s2, we could not analyse the association between *cagA* status and *vacA* genotypes and between *iceA* and *vacA* genotypes (Table III).

### Relationship between genotypes and gastric diseases

Of the 197 strains studied, 86 were diagnosed with gastric ulcer, 58 with gastritis and 53 with gastric cancer. *VacA* s1cm2 was detected in all the disease conditions, and it was more significantly associated with the presence of gastric cancer (p<0.05). S1am2 and s1cm1 were detected in all the disease except gastric cancer, while s1bm2 was found in gastric cancer alone (Table IV). Surprisingly, *iceA1* had a statistically significant association with gastric cancer (p<0.05). Neither *cagA* nor *iceA2* was associated with various diseases. The most prevalent combination *cagA*/s1cm2/*iceA1* was present in 56.6% (95 of 168) including 58.0% (40 of 69) of gastric ulcer, 47.0% (23 of 49) of gastritis and 64.0% (32 of 50) of gastric cancer (Table V). However, no significant association was found between the combination genotypes and diseases (p>0.05).

## Discussion

This study was designed to characterize the genotype of *H. pylori* from gastric biopsy specimens from patients with upper gastrointestinal diseases and the relationship with clinical outcome in Northeast China. *H. pylori* was analysed for the presence of the genes for *cagA*, *vacA* and *iceA*. To our knowledge, this was the first study to analyse the different proposed virulence genes characterized in *H. pylori* and the relationship between the genes and upper gastrointestinal diseases in Northeast China.

*CagA* gene, as a major *H. pylori* virulence factor, was reported to be strongly associated with atrophic gastritis and gastric cancer as previously described. This is probably the main cause of a high incidence of gastric cancer in the region of East Asia, where the percentage of *cagA*-positive strains is above 90% (12). Worldwide, the presence of the *cagA* gene varies from 50% in some Middle Eastern countries to 99% in East Asian countries (13–15). In this study, *cagA* was found in 89.3% of *H. pylori*-infected patients. The result is similar to data reported from other districts of China (11,16). However, we did not find an association between *cagA* and clinical results. Notably, of the 29 mixed infection cases, 8 had *cagA-*negative and strains with an absence of *cagA* appeared to be associated with mixed infection (p=0.004).

The present study demonstrated that all strains of *H. pylori* carried the *vacA* s1 allele. Previous studies noted that s1c was present exclusively in isolates from East Asia (16–18). Our report also demonstrated a high prevalence of type s1c strains in this region, up to 94.4%. The result was similar to the report of Wang *et al* (19) and slightly higher than the prevalence in Beijing and Shanghai, which may result from the fact that more foreigners from America and Europe live in the two cities above, as either the s1a or s1b subtype was present in almost all strains in Central and South America, and in the majority of strains in Spain and Portugal (20,21), nevertheless rarely in East Asia (12,16). The *vacA* s2 genotype was prominently prevalent in Africa (9), and consistent to the outcome reported from China and Korea, s2 failed to be detected in this study (19,22).

Worldwide prevalence of *vacA* strains varies geographically. S1m1 strains were predominant in Japan and Korea (18,23) while s1m2 was found in Turkey and Northern and Eastern Europe (20,24). In Alaskans, *H. pylori* had either the *vacA* s1m1 (44.6%) or s2m2 (38.3%) (15). In China, prevalence of strains documented a greatly distinct pattern, with s1m1 and s1m2 sharing the same proportion in the Province of Xi’an (25) and s1m2 strains in Beijing, Taiwan and Hong Kong (16,19,26). The latter condition was similar to our study.

Generally, s1m2 forms of *vacA* bind to and vacuolate a narrower range of cells than s1m1 forms and induce less damage, yet they also act as efficient membrane pores and increase paracellular (27) permeability. The alleles of s1m1 and s1m2 encode to produce toxin which are common in patients with gastrosis (27). In Latin America and Germany, s1m1 was found to have a high correlation with gastric ulcer and gastric carcinoma (21,28). The strains of *vacA* s2m1 and s2m2 engender low toxic toxin which rarely correlates with gastric ulcer and gastric carcinoma (29). In our study, the *vacA* gene encoding the s1cm2 was associated with gastic cancer. Therefore, the s-region should be responsible for gastrosis other than the m-region.

Another virulent factor is the *iceA* gene, with two allelic variants *iceA1* and *iceA2* having been identified. The prevalence of the *iceA1* genotype is 70.1% in this study, basically consistent with data reported from China, Thailand, Korea and Tunisia (9,23,30,31). Meanwhile, *iceA2* is predominant in Brazil, the US, Europe and South Africa (6,18,32,33). It was demonstrated that *iceA1* was significantly associated with peptic ulcer disease in Holland (34) and the US (35). However, studies from other countries such as in Korea, Colombia and India could not confirm the result (18,36). Some researchers found that the *iceA2* genotype was most frequently found in patients with duodenal ulcer or gastric carcinoma (18,36). However, it is difficult to admit that *iceA2*, a gene that is considered as a protective factor in some regions and that is associated with more severe diseases in other places, could be considered a molecular marker of more virulent *H. pylori* strains (33). It was well worth mentioning that the *iceA1* strains, based on this study, have a significant association with gastric cancer.

In common with other studies, there exists a strong indication that the presence of multiple *H. pylori* strains are detectable in clinical samples. Some studies have claimed multiple genotypes have a link with duodenal ulcers (9). It may be speculated that multiple strains contribute to increasing the potential chances of infecting pathogen. By colonizing a variety of receptors expressed on gastric epithelial cells, m1 and m2 strains probably tend to bring about pathological changes. Multi-colonization arising from the co-existence of more than one strain exert burden to patients under eradication treatment and furthermore dramatically enhance the risk of malignant tumors of the digestive tract among adult patients. However, our data did not indicate that multiple strain infection increases the risk of developing diseases (p>0.05).

In conclusion, the present study identified the prevalence of main virulence factor genes *cagA*, s1cm2 and *iceA1* in Northeast China. The *vacA* gene encoding s1cm2 was found to predominate in gastic cancer patients, and the *iceA1* genotype was also associated with gastric cancer. It may be insufficient to analyse gastrointestinal diseases simply by genotyping *H. pylori*, and therefore, we must evaluate the pathogenesis of diseases by a combination of the analysis of bacterial factors, genetic factors of the host and environmental factors.

## Figures and Tables

**Table I:** Primer sequences for human HP 16S rRNA, cagA, vacA and iceA.

Gene	Primer	Primer sequence (5′→3′)^a^	Product size (bp)	Reference
16S rRNA	cp-1	GCGCAATCAGCGTCAGGTAATG	500	(37)
	cp-2	GCTAAGAGATCAGCCTATGTCC		
cagA	cagA-F	GATAACAGGCAAGCTTTTGAGG	349	(18)
	cagA-R	CTGCAAAAGATTGTTTGGCAGA		
s1a	S1a-F	TCTYGCTTTAGTAGGAGC	212	(18)
	VA1-R	CTGCTTGAATGCGCCAAAC		
s1b	SS3-R	AGCGCCATACCGCAAGAG	187	(18)
	VA1-R	CTGCTTGAATGCGCCAAAC		
s1c	S1c-F	CTYGCTTTAGTRGGGYTA	213	(18)
	VA1-R	CTGCTTGAATGCGCCAAAC		
s2	SS2-F	GCTAACACGCCAAATGATCC	199	(8)
	VA1-R	CTGCTTGAATGCGCCAAAC		
m1	VA3-F	GGTCAAAATGCGGTCATGG	290	(38)
	VA3-R	CCATTGGTACCTGTAGAAAC		
m2	VA4-F	GGAGCCCCAGGAAACATTG	352	(38)
	VA4-R	CATAACTAGCGCCTTGCAC		
iceA1	iceA1-F	GTGTTTTTAACCAAAGTATC	247	(35)
	iceA1-R	CTATAGCCASTYTCTTTGCA		
iceA2	iceA2-F	GTTGGGTATATCACAATTTAT	229/334	(35)
	iceA2-R	TTRCCCTATTTTCTAGTAGGT		

a Y is C or T, R is A or G and S is C or G.

**Table II:** Distribution of 197 patients with different clinical outcomes, according to age and gender.

	Clinical status			
Classification	GU^a^ n=86 (%)	GS^b^ n=58 (%)	GC^c^ n=53 (%)	Total n=197 (%)
Age (years)				
21–30	7 (8.1)	2 (3.5)	0 (0.0)	9 (4.6)
31–40	12 (14.0)	11 (19.0)	4 (7.5)	27 (13.7)
41–50	33 (38.4)	17 (29.3)	17 (32.1)	67 (34.0)
51–60	24 (27.9)	17 (29.3)	16 (30.2)	57 (29.0)
>60	10 (11.6)	11 (18.9)	16 (30.2)	37 (18.7)
Gender				
Male (M)	52 (60.5)	37 (63.8)	34 (64.2)	123 (62.4)
Female (F)	34 (39.5)	21 (36.2)	19 (35.8)	74 (37.6)
M:F	1:0.7	1:0.6	1:0.6	1:0.6

a Gastric ulcer;

b gastritis;

c gastric cancer.

**Table III:** Association of *vacA* with *cagA* and *iceA* genotypes.

vacA	cagA^+^ n (%)	cagA^−^ n (%)	iceA1 n (%)	iceA2 n (%)	iceA1/iceA2 n (%)
s-region					
s1a	4 (66.7)	2 (33.3)	5 (83.3)	1 (16.7)	0 (0.0)
s1b	3 (60.0)	2 (40.0)	5 (100.0)	0 (0.0)	0 (0.0)
s1c	169 (90.9)	17 (9.1)	128 (68.8)	45 (24.2)	13 (7.0)
m-region					
m1	18 (90.0)	2 (10.0)	14 (70.0)	6 (30.0)	0 (0.0)
m2	139 (93.0)	11 (7.0)	110 (73.4)	38 (25.3)	2 (1.3)
m1m2	19 (84.0)	8 (16.0)	14 (70.4)	2 (22.2)	11 (7.4)
s/m region					
s1am2	4 (66.7)	2 (33.3)	5 (83.3)	1 (16.7)	0 (0.0)
s1bm2	3 (60.0)	2 (40.0)	5 (100.0)	0 (0.0)	0 (0.0)
s1cm1	18 (90.0)	2 (10.0)	14 (70.0)	6 (30.0)	0 (0.0)
s1cm2	132 (95.0)	7 (5.0)	100 (71.9)	37 (26.6)	2 (1.5)
s1cm1m2	19 (70.4)	8 (29.6)	14 (51.9)	2 (7.4)	11 (40.7)

**Table IV:** *vacA*, *cagA* and *iceA* status of *H. pylori* from 197 patients.

	Clinical status			
Genotype status	GU^a^ n=86 (%)	GS^b^ n=58 (%)	GC^c^ n=53 (%)	Total n=197 (%)
vacA				
s1am2	4 (4.6)	2 (3.4)	0 (0.0)	6 (3.0)
s1bm2	0 (0.0)	0 (0.0)	5 (9.4)	5 (2.5)
s1cm1	16 (18.6)	4 (6.9)	0 (0.0)	20 (10.2)
s1cm2	51 (59.4)	43 (74.2)	45 (84.9)^d^	139 (70.6)
s1cm1m2	15 (17.4)	9 (15.5)	3 (5.7)	27 (13.7)
cagA				
cagA^+^	78 (90.7)	53 (91.4)	45 (84.9)	176 (89.3)
cagA^−^	8 (9.3)	5 (8.6)	8 (15.1)	21 (10.7)
iceA				
iceA1	63 (73.3)	34 (58.6)	41 (77.4)^d^	138 (70.0)
iceA2	14 (16.3)	21 (36.2)	11 (20.8)	46 (23.4)
iceA1/iceA2	9 (10.4)	3 (5.2)	1 (1.9)	13 (6.6)

a Gastric ulcer;

b gastritis;

c gastric cancer.

d P<0.05.

**Table V:** Combined *vacA*, *cagA*, *iceA* genotypes.

	Clinical status			
Combination	GU^a^ n (%)	GS^b^ n (%)	GC^c^ n (%)	Total n (%)
s1am2/cagA^+^/iceA1	1 (1.5)	2 (4.1)	0 (0.0)	3 (1.8)
s1am2/cagA^−^/iceA1	2 (2.9)	0 (0.0)	0 (0.0)	2 (1.2)
s1am2/cagA^+^/iceA2	1 (1.5)	0 (0.0)	0 (0.0)	1 (0.6)
s1bm2/cagA^+^/iceA1	0 (0.0)	0 (0.0)	3 (6.0)	3 (1.8)
s1bm2/cagA^−^/iceA1	0 (0.0)	0 (0.0)	2 (4.0)	2 (1.2)
s1cm1/cagA^+^/iceA1	10 (14.3)	2 (4.1)	0 (0.0)	12 (7.1)
s1cm1/cagA^−^/iceA1	2 (2.9)	0 (0.0)	0 (0.0)	2 (1.2)
s1cm1/cagA^+^/iceA2	4 (5.8)	2 (4.1)	0 (0.0)	6 (3.6)
s1cm2/cagA^+^/iceA1	40 (58.0)	23 (47.0)	32 (64.0)	95 (56.5)
s1cm2/cagA^−^/iceA1	2 (2.9)	1 (2.0)	2 (4.0)	5 (3.0)
s1cm2/cagA^+^/iceA2	6 (8.7)	18 (36.7)	11 (22.0)	35 (20.8)
s1cm2/cagA^−^/iceA2	1 (1.5)	1 (2.0)	0 (0.0)	2 (1.2)
Total	69 (100)	49 (100)	50 (100)	168 (100)

a gastric ulcer;

b gastritis;

c gastric cancer. Twenty-nine patients with mixed infection were excluded.
